# Comparative analysis of clinical features, methylation and immune microenvironment in pediatric and adult papillary craniopharyngiomas: results from a multicenter study

**DOI:** 10.1038/s41598-025-12662-8

**Published:** 2025-07-28

**Authors:** Ning Luo, Yi Lin, Jing Feng, Linbo Cai, Yongli Zhang, Xingfu Wang, Wenzhong Mei, Hao Li, Bei Liu, Xueling Qi, Zhixiong Lin

**Affiliations:** 1 Department of Neurosurgery, Sanbo Brain Hospital, Capital Medical University, Beijing, 100093 China; 2 Department of Pathology, Sanbo Brain Hospital, Capital Medical University, Beijng, 100093 China; 3 Department of Oncology, Guangdong Sanjiu Brain Hospital, Guangzhou, 510510 China; 4 Department of Neurosurgery, Kunming Sanbo Brain Hospital, Kunming, 650199 China; 5 Department of Pathology, The First Affiliated Hospital of Fujian Medical University, Fuzhou, 350004 China; 6 Department of Neurosurgery, The First Affiliated Hospital of Fujian Medical University, Fuzhou, 350004 China; 7 Department of Neurosurgery, Children’s Hospital of Fudan University, Shanghai, 201102 China

**Keywords:** Papillary craniopharyngioma, Pediatric, Adult, Diagnosis, Pathophysiology, Diseases, Oncology

## Abstract

Papillary craniopharyngioma (PCP) was previously believed to occur only in adults. Sporadic pediatric PCP (PPCP), confirmed by detection of the BRAF V600E mutation, has been reported since 2018. The differences between PPCP and adult PCP (APCP) remain unclear. This comprehensive study aimed to enhance understanding of PPCP and elucidate the distinctions between PPCP and APCP. We conducted a systematic analysis of cases of PPCP and APCP, focusing on BRAF V600E mutation, methylation profiles, histology, cell proliferation, imaging features, immune microenvironment, and prognosis. This study compiled 17 cases of PPCP from 6 medical centers, constituting 3.11% of the total 546 PCP cases. Both PPCP and APCP cases exhibited the BRAF V600E mutation with no differences in methylation patterns. However, imaging showed PPCP was predominantly cystic and located beneath the diaphragma sellae with some progression to the sella. APCP typically manifested as solid lesions, primarily located in the suprasellar region, inferior to the third ventricle. PPCP histologically displayed extensive inflammatory cellular infiltration, suppurative change, and occasional small granular calcifications, whereas APCP was largely devoid of calcifications. Compared to APCP, PPCP exhibited significantly lower PD-L1 (an immune checkpoint protein expressed on tumor cells) expression and higher expression levels of CD38 (an immunosuppressive marker), S100A8/A9 (a neutrophil marker), and MPO (myeloperoxidase, a neutrophil marker). Additionally, stromal expression of CD163 (an M2 macrophage marker) was lower in PPCP compared to APCP, while CD68 (an M1 macrophage marker), CD3 (T cell marker), and CD20 (B cell marker) expression showed no significant difference. Ki-67 expression exhibited a banded pattern on PPCP epithelium, contrasting with a punctate pattern in APCP, with significantly higher levels observed in PPCP. Postoperative prognosis did not differ between PPCP and APCP. While driver genes and methylation profiles may not differentiate PPCP from APCP, substantial discrepancies exist in tumor location, histology, imaging features, cell proliferation, and immune microenvironment.

**Supplementary Information:**

The online version contains supplementary material available at 10.1038/s41598-025-12662-8.

## Introduction

Craniopharyngioma (CP) represents an epithelial neoplasm localized within the sellar or parasellar region, arising from remnants of the ectoderm and Rathke’s cleft cyst (RCC). The recent fifth edition of the WHO Classification of Tumors of the Central Nervous System has brought forth significant updates in the categorization of CP. The classification now discerns adamantinomatous craniopharyngioma (ACP) and papillary craniopharyngioma (PCP) as distinct tumor entities rather than mere subtypes^1^. PCP is characterized by its association with the activation of the mitogen-activated protein kinase signaling pathway, predominantly instigated by the BRAF V600E mutation occurring in somatic cells^2,3^. Therefore, the identification of the BRAF V600E mutation serves as a pivotal criterion in the diagnosis and differential diagnosis of PCP^2^. PCP primarily manifests in adults, with sporadic occurrences observed in pediatric populations. In 2018, Schlaffer et al. documented an instance of PCP in a 6-year-old child, wherein the presence of the BRAF V600E mutation was detected. This case underscored the potential occurrence of PCP in pediatric patients and hinted at a plausible transformation from RCC^4^. In 2019, Borrill et al. presented a case of PCP involving a 4-year-old child, coupled with a comprehensive literature review^5^. Their review revealed a total of 23 documented cases of pediatric PCP (PPCP) across 6 studies prior to their report. However, among these cases, only the one reported by Schlaffer et al. underwent testing for the BRAF V600E mutation^4^while the remaining cases lacked such genetic analysis^5–7^. Furthermore, a substantial proportion of these cases (18 out of 23) lacked specific clinical details, with descriptions limited to generic mentions of pediatric patients without delineating age ranges^5–8^. Moreover, none of these reports elucidated the disparities in PCP presentation between pediatric and adult populations. In 2021, Takagi et al. reported a case of PCP featuring an intratumoral abscess in a 14-year-old child^9^. The imaging characteristics of this case notably revealed a noncalcified and irregularly enhanced cystic mass. This stands in contrast to the typical presentation of adult PCP (APCP), which often manifests as solid changes predominantly located in the suprasellar region, with enhancement observed in the main body of the tumor^9^. Most APCP are solid changes, mostly located in the suprasellar region, and the tumor parenchyma may show enhancement^9^. In 2022, our research team reported the clinical characteristics of 5 cases of PPCP. Notably, our study introduced a novel diagnostic and therapeutic approach for PPCP^10^. We asserted that PPCP can be accurately and reliably identified through magnetic resonance imaging (MRI) and computed tomography (CT), and additionally observed significant inflammatory changes in the tumor microenvironment, unfortunately, no comparative study with APCP was conducted in this report.

Pediatric brain tumors diverge significantly from their adult counterparts, necessitating a thorough examination of the disparities between PPCP and APCP. In this study, we collected data from 17 cases of PPCP (≤ 14 years)^11^ across 6 medical centers, including the five cases we previously reported^10^.and systematically analyzed their differences from APCP concerning the BRAF V600E mutation, methylation profiles, histological characteristics, cell proliferation, imaging features, immune microenvironment, and prognostic outcomes. This comprehensive approach aimed to enhance comprehension of PPCP and illuminate the distinctions between PPCP and APCP.

## Materials and methods

### Statement

All methods were performed in accordance with the relevant guidelines.

### Study subjects

A retrospective cohort of 17 PPCP patients were assembled from 6 medical centers before June 2022, comprising 10 cases from Sanbo Brain Hospital of Capital Medical University, 2 cases from Fujian Sanbo Funeng Brain Hospital, 1 case from Kunming Sanbo Brain Hospital, 2 cases from Sanjiu Brain Hospital, 1 case from Children’s Hospital of Fudan University, and 1 case from the First Affiliated Hospital of Fujian Medical University. For comparative analysis, 86 APCP patients were selected from a cohort of 399 adult patients who underwent surgery for PCP at Sanbo Brain Hospital, Capital Medical University between April 14, 2004, and August 4, 2022. Ethical approval was granted by the Ethics Committee of Sanbo Brain Hospital, Capital Medical University (No. SBNK-YJ-2023-035-01). All included patients provided signed informed consent to participate in the study.

The enrollment criteria for PPCP included individuals aged 14 years or younger, with a confirmed pathological diagnosis of PCP and positive BRAF V600E mutation status. Conversely, APCP patients were defined as those older than 14 years, with a pathological diagnosis of PCP and positive BRAF V600E mutation.

### H&E staining and immunohistochemistry

Postoperative specimens from all cases underwent fixation in neutral formalin and subsequent embedding in paraffin wax. Histological and immunohistochemical analyses were conducted on these surgical specimens. Standard H&E staining and immunohistochemistry were carried out using primary antibodies targeting BRAF V600E, S100A8/A9, S100A9, PD-1, PDL1, Ki67, β-catenin, MPO, CD3, CD20, CD38, CD68, and CD163. Staining evaluation was performed through observation under a high-resolution microscope, following previously established protocols^3,10^. Detailed regarding the reagents and their respective operating concentrations was presented in Table 1.

**Table 1:** Details of the antibody and reagents used in this study.

Name of antibodies	Model	Norm	Factory owners	Operating concentration	Host species
Anti-S100A9 + Calprotectin(S100A8/A9 complex) antibody	ab22506	10 µg	Abcam	1 µg/ml (1/1000)	Mouse
Anti-PD-L1 antibody	ab213524	40 µl	Abcam	1/250	Rabbit
CD38 Antibody Reagent (immunohistochemistry)	MAB-0755	0.2 ml/bottle	Maixin	1/100–1/200	Mouse
CD163 Antibody Reagent (immunohistochemistry)	MAB-0869	0.2 ml/bottle	Maixin	1/100–1/200	Mouse
PD-1 Antibody Reagent (immunohistochemistry)	MAB-0734	0.2 ml/bottle	Maixin	1/100–1/200	Mouse
Myeloperoxidase antibody reagent (immunohistochemistry)	RAB-0379	0.2 ml/bottle	Maixin	1/50–1/100	Rabbit
BRAF V600E	–	5 ml	Roche	12 µg/ml	Mouse
Ki67	ZM-0167	0.2 ml/vial	Zhongshan Golden Bridge	1/200	Mouse
CD3	ZM-0417	3 ml/vial	Zhongshan Golden Bridge	Working solution	Mouse
CD20	ZM-0039	3 ml/bottle	Zhongshan Golden Bridge	Working solution	Mouse
CD68	ZM-0060	3 ml/vial	Zhongshan Golden Bridge	Working solution	Mouse
β-catenin	ZM-0442	3 ml/vial	Zhongshan Golden Bridge	Working solution	Mouse

Immunohistochemical quantification was conducted according to established guidelines^12^ staining procedures can be found in Supplementary 1. Positivity intensity was graded on a scale ranging from 0 to 3, with strong positivity designated as 3, positive as 2, weak positive as 1, and negative as 0. Additionally, the percentage of positive cells was assessed, with scores ranging from 0 to 5 points. A score of 0 indicated 0% positive cells, while scores of 1, 2, 3, 4, and 5 corresponded to 0–20%, 20–40%, 40–60%, 60–80%, and 80–100% positive cells, respectively. In a single field of view, the percentage of positive tumor cells represents the ratio of positive tumor cells to all tumor cells observed. Similarly, the percentage of positive stromal cells denotes the proportion of positive tumor cells among all stromal cells present. The final score is calculated as the product of the positive intensity score and the percentage of positive cells. Five fields of view at 20x magnification were chosen from an immunohistochemical image, and the scores from these 5 fields were averaged. All scores were recorded and calculated for tumor parenchyma and stroma, respectively.

### Methylation analysis

Of the 17 collected PPCP cases, 15 underwent formalin fixation and paraffin embedding for methylation analysis. Among these, 7 cases were recurrent tumors and 8 cases were primary tumors, all exhibiting positivity for BRAF V600E. Additionally, 15 APCP cases, aged 58–71 years, were sourced from the formalin-fixed and paraffin-embedded specimen bank of Sanbo Brain Hospital of Capital Medical University as the reference. Within this group, 3 cases were recurrent tumors and 12 were primary tumors. DNA methylation profiling was conducted using the Illumina Infinium Human Methylation 850 (850k) BeadChip platform (Illumina, San Diego, USA).

DNA extraction was performed on formalin-fixed paraffin-embedded tumor samples. Samples exhibiting a CpG site detection rate (detection p-value < 0.05) exceeding 95% were included for further analysis. However, one PPCP sample was excluded due to a CpG site detection rate below 95%. Subsequently, data underwent preprocessing and normalization using the minfi software, with calculation of beta and M values.

Probes meeting the following criteria were excluded from subsequent analyses: (1) probes with detection p-values exceeding 0.01, where detection p-values were computed based on the total signal (M + U) per probe relative to the background signal level estimated from negative control probes; (2) probes targeting genes located on the X and Y chromosomes; (3) probes containing single nucleotide polymorphism (SNP) 147; and (4) probes mapped to multiple genomic locations. Consequently, a total of 657, 541 probes remained eligible for analysis^13^.

### Patient treatment status

Among the 17 pediatric cases, 10 underwent complete resection, of which 2 recurred and 8 did not. In the remaining 7 cases with incomplete resection, all regrowth. Among the 86 adult patients, 52 had complete resection, with 6 recurring and 46 not recurring. Of the remaining 34 patients with incomplete resection, 31 recurred and 3 did not. Among the patients included in this study, only one pediatric patient received targeted therapy, and two adult patients underwent Gamma Knife radiosurgery.

### Statistical analysis

Data validation procedures were employed to ensure the integrity and accuracy of the dataset, involving the removal of missing values and rectification of data errors and anomalies. Subsequently, descriptive statistics such as mean, frequency, and percentage were computed to delineate the basic characteristics and distribution of the samples. Comparative analyses between pediatric and adult groups were conducted utilizing the two-sample *t*-test to compare continuous variables, and performed FDR multiple testing corrections. Spearman’s rank correlation coefficients were employed to evaluate the correlation between clinical presentations, imaging features, and histopathological findings. Chi-square tests were used to compare the differences in imaging characteristics between pediatric and adult patients, as well as the prognostic outcomes between complete resection and incomplete resection. Survival curve analysis for pediatric and adult patients was performed using the log-rank (Mantel-Cox) test and the Gehan-Breslow-Wilcoxon test. All statistical analyses were conducted using GraphPad Prism 8.

## Results

### Patient characteristics and differences in imaging features

**Fig. 1:**
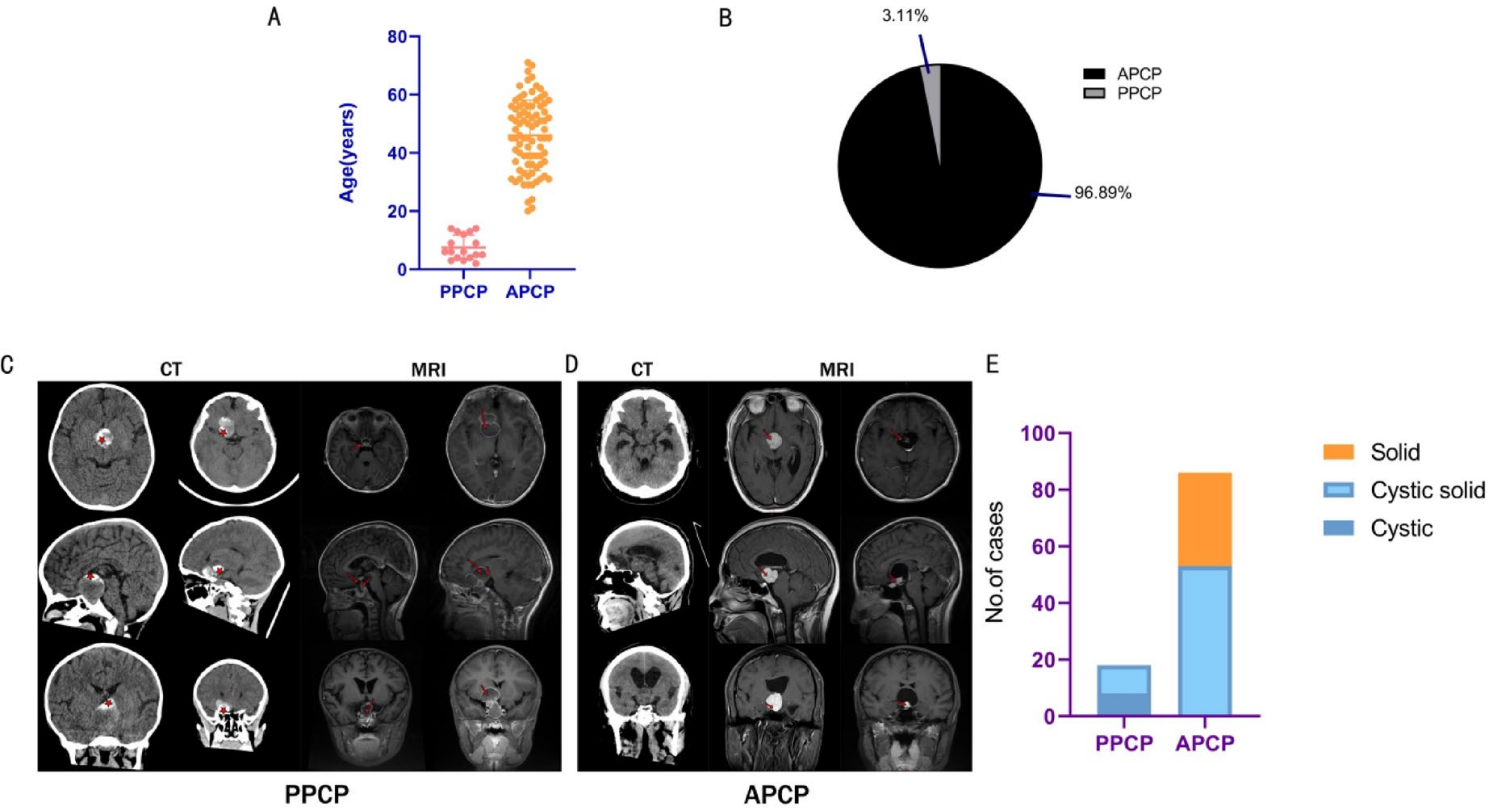
Patient characteristics and differences in imaging features. (**A**) Age distribution: 7.5y (pediatric) vs. 46.1y (adult). (**B**) Case composition: 17 pediatric vs. 529 adult PPCP. (**C** and **D**) Imaging contrast: PPCP predominantly infradiaphragmatic cystic lesions (star: sand-like calcification) vs. APCP suprasellar solid masses (red arrow: homogeneous enhancement). (**E**) Significant cyst-solid pattern divergence between groups ( χ2 test, *P* < 0.01).

This study encompassed 17 cases of PPCP from 6 medical centers. The mean age of the pediatric cohort was 7.5 years (Fig. 1A), constituting 3.11% of the total 546 PCP cases admitted to these 6 centers during the study period (Fig. 1B). Eighty-six cases of APCP, admitted to Sanbo Brain Hospital of Capital Medical University before June 2022, were randomly sampled, with a mean age of 46.1 years (Fig. 1A) (Table 2).

**Table 2:** Demographics and baseline characteristics.

	Pediatric PCP	Adult PCP
Number of patients	17	86
Mean age (year)	7.5	46.1
Sex		
Male	10 (58.8%)	58 (67.4%)
Female	7 (41.2%)	28 (32.6%)
Status		
Primary tumor	9 (52.9%)	52 (60.5%)
Recurrent tumor	8 (47.1%)	34 (39.5%)
Patients undergoing DNA methylation analysis	15	15

PCP, papillary craniopharyngioma.

In the majority of PPCP, tumors were typically located beneath the diaphragma sellae, often presenting as cystic formations with some displaying a combination of cystic and solid components. Cystic walls were enhanced. The tumors exerted pressure, displacing the diaphragma sellae upward towards the base of the third ventricle. No hydrocephalus was observed. The pituitary gland was frequently obscured or not visibly discernible. CT imaging revealed scattered sand-like hyperintense signals within the tumor, with no evidence of eggshell-like calcifications (Fig. 1C).

In most cases of APCP, tumors were commonly situated entirely within the suprasellar region, anterior to the third ventricle, exhibiting a round-like shape and solid consistency. The pituitary gland below remained distinctly visible. Contrast imaging showed homogeneous enhancement. No calcifications were observed on CT scans. In a minority of cases, cystic changes were evident. Tumor parenchyma typically presented as hypointense or isointense on CT scans, with isointensity on T1-weighted MRI scans, while signal intensity on T2-weighted scans varied (Fig. 1D). There was a significant difference in cystic-solid presentation between PPCP and APCP (Fig. 1E). Statistical analysis showed significant difference in cystic-solid presentation between PPCP and APCP (χ2 = 45.22, *p* < 0.01).

We also found that there were no significant differences in imaging characteristics, including calcification, cystic-solid architecture, and enhancement patterns, between primary and recurrent PPCP. Intraoperatively, these tumors demonstrated moderate vascularity without clear evidence of invasion but were often found to be tightly adherent to adjacent structures. The solid tumor tissue was typically soft and amenable to aspiration. However, in cases of recurrent PPCP, the presence of scar tissue was observed, though it still did not show significant invasive behavior towards surrounding tissues.

### Difference in histology and proliferation between PPCP and APCP

**Fig. 2:**
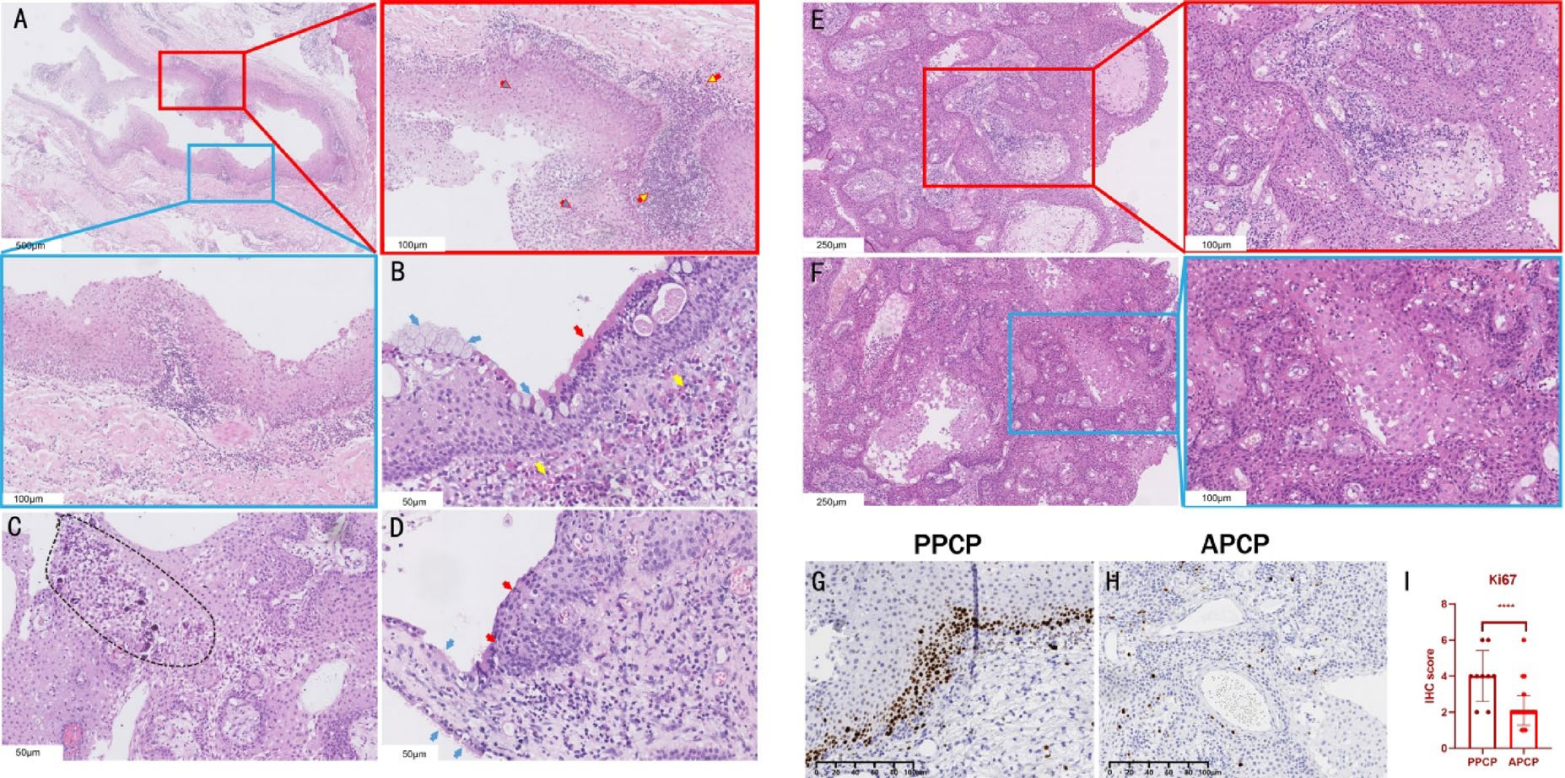
Difference in histology and proliferation between PPCP and APCP. (**A**–**D**) PPCP-specific histology: Pan-layer inflammatory infiltration (A), mucin-ciliated epithelial complexes (B arrows), calcification (C), Rathke’s cyst epithelial migration (D arrows). (**E** and **F**) APCP representative H&E. (**G** and **H**) Ki-67 spatial dichotomy: Band-like distribution (PPCP) vs. scattered dot-like pattern (APCP). (**I**) Quantified proliferative disparity (t = 6.146, *P* < 0.01). Staining markers: Blue arrow-mucin vacuoles, Red arrow-ciliated epithelium, Yellow arrow-pheochromocyte-like cells.

The cyst structure of PPCP exhibited more prominence than that of APCP in histological test, with flattened papillae resembling the squamous cell epithelium of RCC, where ciliary epithelium encapsulates the cystic surface. Various inflammatory cell infiltration and suppurative changes were frequently noted in PPCP, with calcifications being relatively rare and typically manifesting as fine granular formations. In recurrent PPCP, an increased presence of stromal tissue was often observed, typically forming cord-like scar structures that separated the tumor parenchyma. However, the parenchymal architecture of recurrent PPCP showed no significant differences compared to that of primary PPCP. No calcifications were observed in APCP (Fig. 2A–F).

In PPCP epithelium, Ki-67-positive cells demonstrated a band-like distribution (Fig. 2G), whereas in APCP epithelium, they exhibited scattered dot-like pattern (Fig. 2H). Ki-67 expression was significantly higher in PPCP compared to APCP (Fig. 2I). Immunohistochemical quantification indicated significantly different pattern between PPCP and APCP (*t* = 6.146, *p* < 0.01).

### Difference in BRAF V600 mutation and methylation between PPCP and APCP

**Fig. 3:**
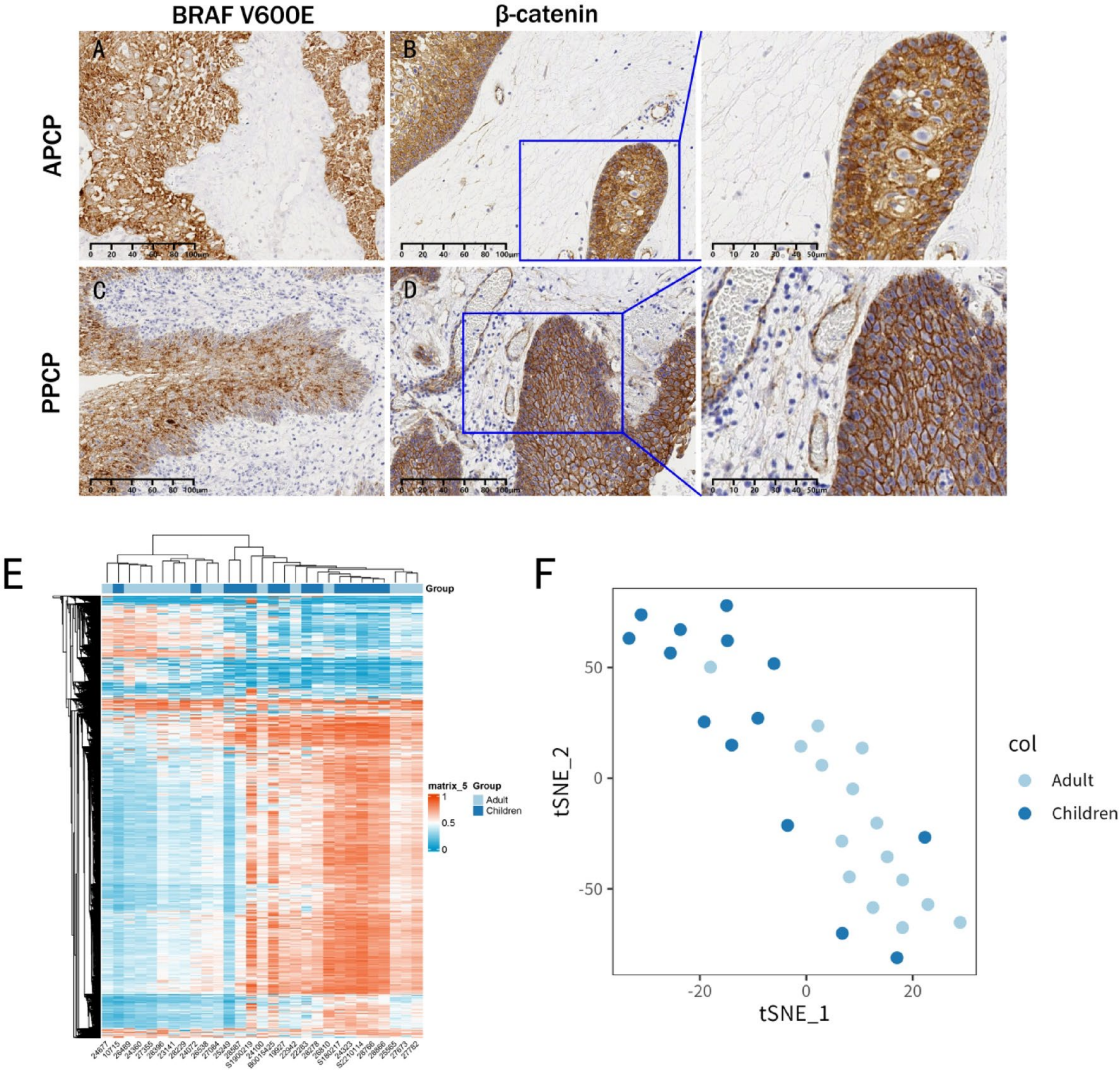
BRAF V600E, β-catenin, and methylation profiles in PPCP and APCP. (**A**, **C**) Parenchymal BRAF V600E positivity (stroma-negative) in both groups. (**B**, **D**) Pan-cytoplasmic β-catenin expression (nuclear-negative). (**E**, **F**) Hierarchical clustering analysis and t-stochastic neighbor embedding analysis revealed no significant difference in the methylation profiles between PPCP and APCP.

In both PPCP and APCP, BRAF V600E was expressed in the cytoplasm of tumor cells, with no significant difference in expression between the two groups (Fig. 3A, C). No significant alterations in the BRAFV600E status were found between the 9 cases of primary PPCP and the 8 cases of recurrent PPCP.

β-catenin exhibited nearly 100% positive expression in the tumor cell membranes but was negatively expressed in the nucleus. Furthermore, β-catenin was not expressed in the stroma in either PPCP or APCP (Fig. 3B, D). No disparity in β-catenin expression was observed between PPCP and APCP.

Unsupervised hierarchical clustering utilizing 10, 000 probes with the highest methylation differences and t-stochastic neighbor embedding analysis were conducted using the ComplexHeatmap and Rtsne suite of software in R 4.0. The hierarchical clustering analysis (Fig. 3E) and the t-stochastic neighbor embedding analysis (Fig. 3F) did not reveal significant differences in methylation profiles between PPCP and APCP.

### Difference in immune microenvironment between PPCP and APCP

**Fig. 4:**
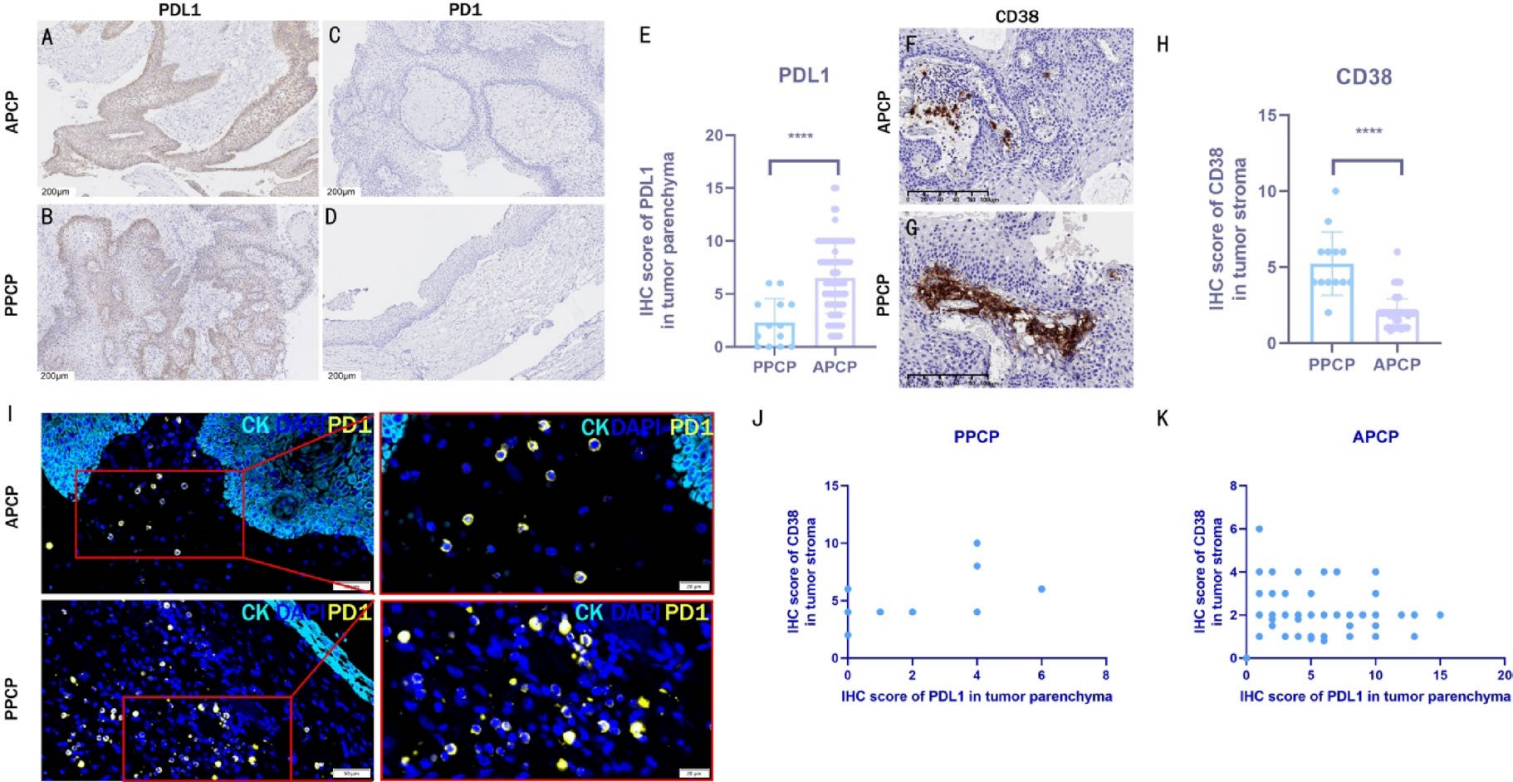
PD-L1, PD-1 and CD38 expression profiles in PPCP and APCP. (**A**-**B**) PD-L1 cytoplasmic localization in tumor parenchyma. (**C**-**D**) Absence of PD-1 in parenchyma/stroma. (**E**) Lower PD-L1 expression in PPCP vs. APCP (t = 4.351, *p* < 0.01,). (**F**-**H**) CD38 stromal expression with higher intensity in PPCP vs. APCP (t = 9.452, *p* < 0.01). (I) PD-1 stromal detection by immunofluorescence. (**J**-**K**) Correlation analysis: PPCP (*r* = 0.4433, *p* = 0.1294); APCP (*r*= − 0.2549, *p* = 0.020).

Immunohistochemistry demonstrated that PD-L1 (programmed death-ligand 1, an immune checkpoint protein expressed on tumor cells) was expressed in the tumor parenchyma cells of both PPCP and APCP, while it was absent in the stromal cells (Fig. 4A, B). Semi-quantitative analysis revealed a significantly lower expression of PD-L1 in PPCP compared to APCP (Fig. 4E) PD-L1 expression was significantly lower in PPCP compared to APCP (*t* = 4.351, *p* < 0.01). PD-1 (programmed cell death protein 1, expressed on activated T cells) was not expressed in either the tumor cells or stromal cells of PPCP and APCP (Fig. 4C, D), although PD-1 was detected in the stromal cells of both PPCP and APCP through immunofluorescence staining (Fig. 4I).

CD38-positive cells (an immunosuppressive marker) were exclusively observed in the stroma of both PPCP and APCP, with a significantly higher abundance in PPCP compared to APCP (*t* = 9.452, *p* < 0.01). (Fig. 4F, G, H). No significant correlation between PD-L1 and CD38 was detected in PPCP (*r* = 0.4433, *p* = 0.1294)(Fig. 4J). However, a weak negative correlation was noted in APCP (*r* = − 0.2549, *p* = 0.0200) (Fig. 4K).

**Fig. 5:**
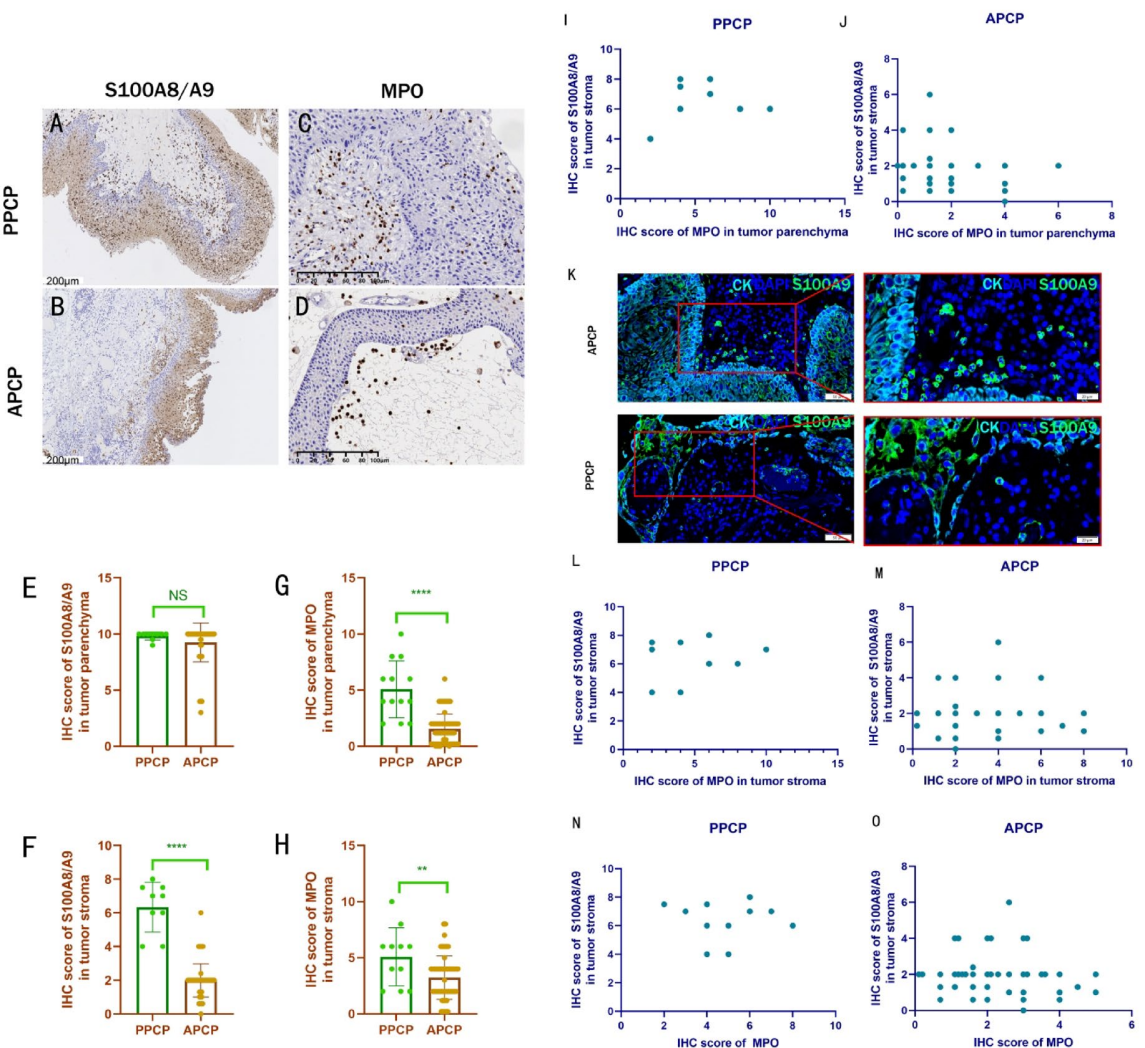
Expression of S100A8/A9 and MPO in PPCP and APCP. (**A**, **B**) Ubiquitous S100A8/A9 expression in tumor parenchyma and stroma. (**C**, **D**) MPO detection in both subtypes. (**E**) Comparable parenchymal S100A8/A9 between groups. (**F**) PPCP exhibited significantly higher S100A8/A9 expression in stromal cells compared to APCP (t = 11.92, *p* < 0.01) (**G**, **H**) PPCP shows elevated MPO in both parenchyma (t = 7.718, *P* < 0.01) and stroma (t = 2.879, *P* < 0.01). (**I**, **J**) No significant correlations between MPO parenchyma and S100A8/A9 stroma in either subtype. (**K**) Immunofluorescence validation of S100A8 spatial distribution. (**L**, **O**) Lack of stromal MPO-S100A8/A9 correlations across compartments (all *P* > 0.05).

S100A8/A9 (S100 calcium-binding protein A8/A9, a neutrophil marker) was expressed in nearly 100% of the non-basal layer of the tumor parenchyma in both PPCP and APCP (Fig. 5A, B). Additionally, MPO (myeloperoxidase, a neutrophil marker), another marker of neutrophils, was expressed in both parenchymal and stromal cells of PPCP and APCP (Fig. 5C, D). Immunohistochemical quantification of S100A8/A9 expression in parenchymal cells showed no difference between PPCP and APCP(*t* = 0.9958, *p* > 0.05). However, there was greater individual variation observed in APCP (Fig. 5E). S100A8/A9 was also expressed in the stoma of both PPCP and APCP, serving as a marker of neutrophils. Immunohistochemical quantification indicated significantly higher S100A8/A9 expression in PPCP compared to APCP (*t* = 11.92, *p* < 0.01)(Fig. 5F), consistent with our previous observation of increased inflammatory infiltration in PPCP under H& staining. Quantitative analysis of MPO expression revealed higher expression levels in both the parenchyma and stroma of PPCP compared to APCP(*t* = 7.718, *p* < 0.01) (*t* = 2.879, *p* < 0.01) (Fig. 5G, H).

As MPO was not expressed on tumor cells, it was classified as neutrophil marker in both the parenchyma and stroma. S100A8/A9, expressed on tumor cells, was considered a neutrophil marker solely in the stroma. In PPCP and APCP, correlation analysis found no significant correlation between MPO parenchymal expression and stromal expression of S100A8/A9(*r* = 0.2135, *p* = 0.5224, *r* = − 0.2517, *p* = 0.0627) (Fig. 5I, J). In both PPCP and APCP, stromal expression of MPO did not correlate with the stromal expression of S100A8/A9, either in PPCP (*r* = 0.01699, *p* = 0.9628) or APCP (*r* = − 0.07209, *p* = 0.5146). (Fig. 5L, M). Furthermore, no correlation was found between total MPO expression and S100A8/A9 expression in the stroma, either in PPCP (*r* = − 0.09199, *p* = 0.7868) or APCP (*r* = − 0.1704, *p* = 0.1211) (Fig. 5N, O). These findings suggested that MPO and S100A8/A9-labeled cells in the stroma may represent different subtypes. Immunofluorescence test clearly demonstrated S100A9 expression in the cytoplasm of various cells within the parenchyma and stroma of PPCP and APCP (Fig. 5K).

**Fig. 6:**
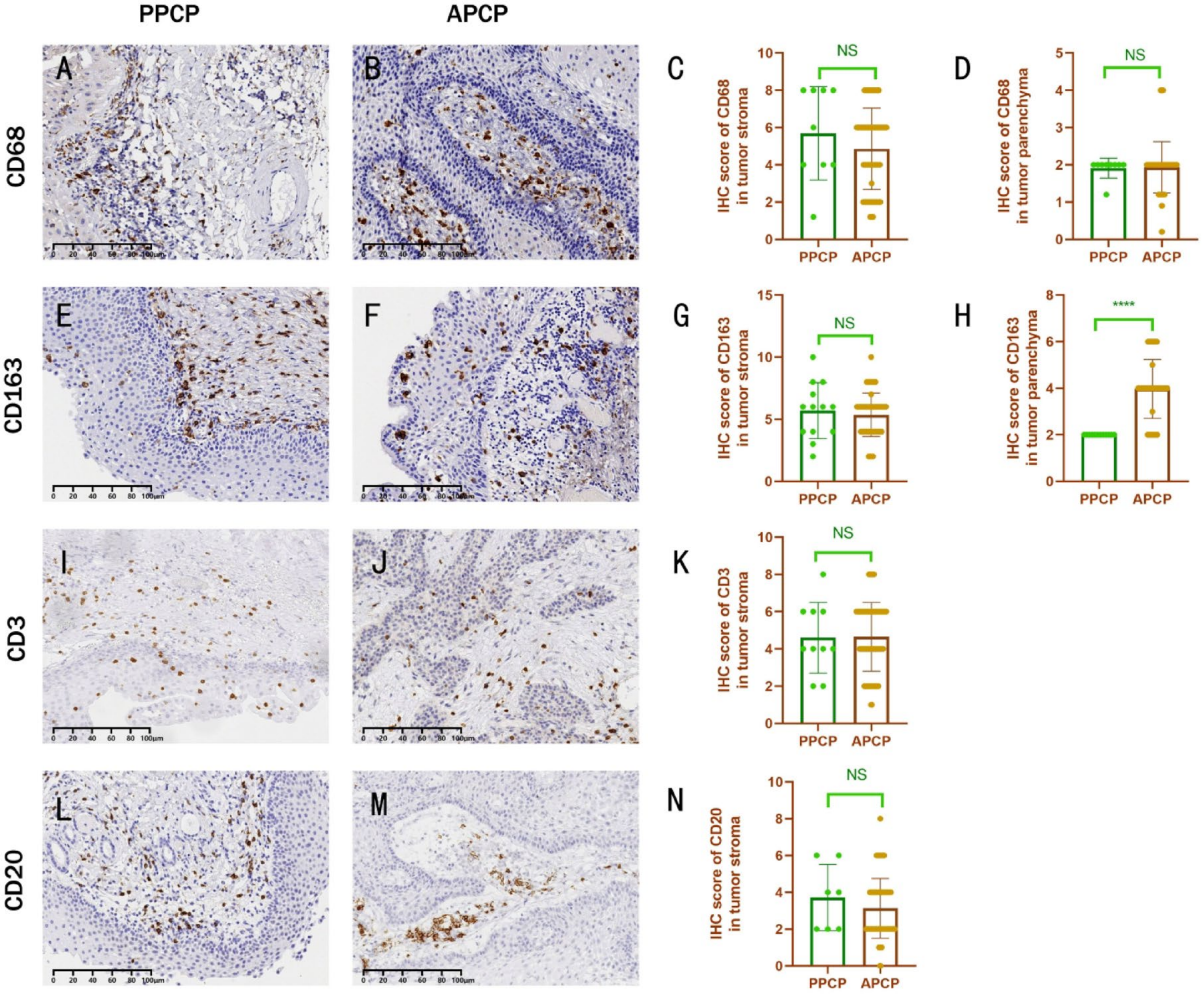
Expression of CD68, CD163, CD3, and CD20 in PPCP and APCP. (**A**, **B**) Stromal-enriched CD68 pattern conserved across subtypes. (**C**, **D**) No intergroup differences in CD68 distribution (parenchyma *P* > 0.05, stroma *P* > 0.05). (**E**, **F**) Bicompartmental CD163 expression. (**G**) Comparable stromal CD163 between groups. (**H**) Significantly higher parenchymal CD163 in APCP vs. PPCP (t = 5.393, *P* < 0.01). (**I**–**N**) Conserved stromal CD3/CD20 expression without intergroup differences (all *P* > 0.05).

CD68 (M1 macrophage marker) and CD163 (M2 macrophage marker) served as markers of macrophages, with CD68-positive cells observed in both the parenchyma and stroma of PPCP and APCP, exhibiting a more pronounced presence in the stroma (Fig. 6A, B). This distribution pattern did not significantly differ between PPCP and APCP, (parenchyma: *t* = 1.065, *p* > 0.05; stroma: *t* = 0.1054, *p* > 0.05) (Fig. 6C, D). Likewise, CD163-positive cells were detected in both the parenchyma and the stroma of PPCP and APCP (Fig. 6E, F). Quantitative analysis revealed higher parenchymal expression of CD163 in APCP than in PPCP(*t* = 5.393, *p* < 0.01) (Fig. 6H). However, there was no significant difference in stromal expression of CD163 between PPCP and APCP (*t* = 0.6062, *p* > 0.05) (Fig. 6G). CD3 (T cell marker) and CD20 (B cell marker) are markers of T cells and B cells, respectively. Both CD3 (Fig. 6I, J, K) and CD20 (Fig. 6L, M, N) were expressed in the stroma of PPCP and APCP, with no significant difference observed in their expression between PPCP and APCP(*t* = 0.08369, *p* > 0.05), (*t* = 0.9170, *p* > 0.05).

**Fig. 7:**
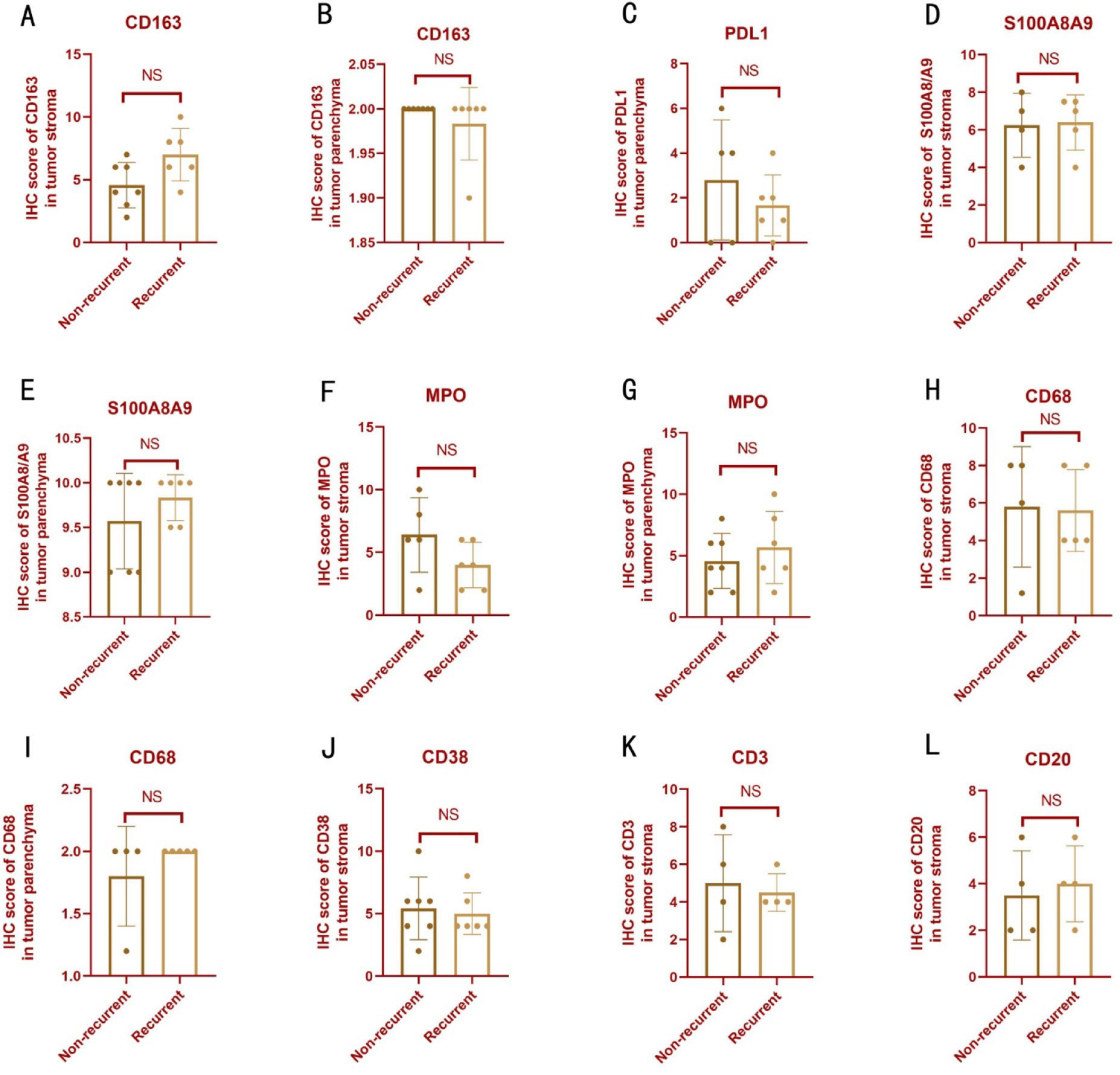
Comparative analysis of immune marker expression between non-recurrent and recurrent PPCP using immunohistochemistry (IHC). (**A**, **B**) Comparable CD163 stromal/parenchymal IHC scores. (**C**) PDL1 parenchymal expression parity. (**D**–**G**) Stable S100A8/A9 and MPO profiles across compartments. (**H**–**J**) Conserved CD68/CD38 stromal distribution. (**K**, **L**) Unaltered stromal CD3/CD20 infiltration.

Furthermore, we specifically compared the indicators of the aforementioned microenvironments between primary and recurrent PPCP. We found that no indicators showed statistically significant differences. (Fig. 7)

### Difference in prognosis and between PPCP and APCP

**Fig. 8:**
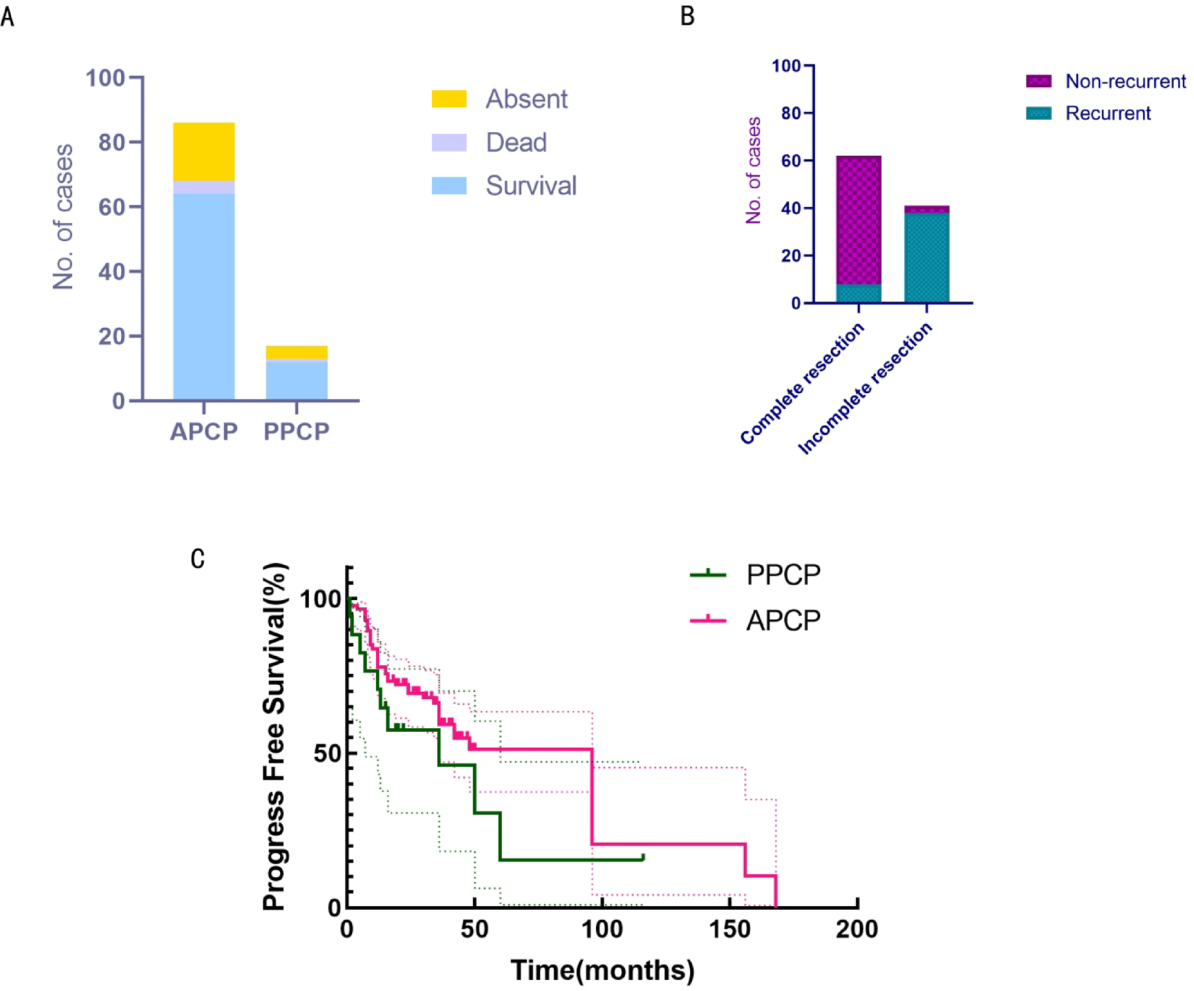
Difference in prognosis between PPCP and APCP. (**A**) Comparable overall prognosis between PPCP and APCP (χ2 test, *P* > 0.05). (**B**) Significant prognostic association with initial resection extent (Fisher’s exact test, *P* < 0.01, total vs. partial). (**C**) Progression-free survival parity post-last surgery (Kaplan–Meier curves, *P* > 0.05).

After a follow-up period ranging from 5 to 12 years, among the 86 APCP patients, 64 patients survived, with 58 remaining recurrence-free, 6 experiencing recurrence, 6 deceased; 16 were lost to follow-up. Among the 17 pediatric patients, 12 survived, 1 passed away; 4 were lost to follow-up (Fig. 8A). Based on survival endpoint events, statistical analysis using the chi-square test showed no significant difference in prognosis between PPCP and APCP (χ^2^ = 0.1174, *p* = 0.9430). Notably, the initial surgical approach, whether total or partial resection for PCP, significantly influenced prognosis(Fisher’s exact test, *p* < 0.01). (Fig. 8B). Furthermore, analyses of progression-free survival subsequent to the last surgery revealed no significant difference between PPCP and APCP (*p* = 0.1768), as assessed by the Log-rank (Mantel-Cox) test and the Gehan-Breslow-Wilcoxon test (Fig. 8C).

## Discussion

This study marks the first comprehensive analysis of the differences between PPCP and APCP as documented in the recorded literature. The present multicenter retrospective study further supports the existence of PPCP, accounting for only 3.11% of all PCP. Currently, the diagnosis and differential diagnosis of PCP is based on histopathologic features and BRAF V600E testing^2,14–20^. Although immunohistochemistry showed positive expression of BRAF V600E cytoplasm in both PPCP and APCP cases and methylation analysis showed no difference between PPCP and APCP, there were still differences in imaging, histology, and immune microenvironment.

Histologically, PCP exhibits well-differentiated stratified squamous epithelium forming papillary structures, accompanied by inflammatory cell infiltration in the stroma. In APCP, calcification is typically absent, with a distinct demarcation between tumor tissue and the hypothalamus^18^. Conversely, in PPCP, the cystic structure predominates over solid components, and papillary structures often appear flattened, resembling the squamous epithelium in RCC, where the squamous epithelial layer encapsulates the cystic surface. Additionally, PPCP may exhibit infiltration of various inflammatory cells, prominent suppurative changes, and occasional small granular calcifications. Our study further corroborated the pronounced inflammatory nature of PPCP, highlighting instances of misdiagnosis as abscesses in the literature^4,6,10^.

On imaging, APCP tumors typically localize to the superior-anterior region of the third ventricle^18,21^ and manifest as solid lesions^18,22^. The solid component of APCP typically exhibits homogeneously enhancement, with calcification being rare^18,22^. In this study, PPCP imaging indicated that lesions primarily located beneath the diaphragma sellae. However, some tumors may breach the diaphragma sellae and ascend, often deviating laterally. CP below the diaphragma sellae often extends into the sella turcica, assuming a round or elliptical shape, accompanied by pituitary fossa enlargement, consistent with previous findings^10,22^. Breakthrough growth in PPCP often assumes a fusiform shape and ascends. Previous literature reported that CT imaging showed sparse intratumoral calcification in PPCP^10^. Conversely, in ACP, calcification typically localizes to the cystic wall or appears as a nodular structure within the tumor^18,23^. While it was previously observed that PPCP cysts increase in size over time without evident solid or cystic changes^10^ several cases in our study exhibited cystic alterations. These characteristics can aid in distinguishing PPCP from RCC in this anatomical region. The cystic wall of RCC typically does not enhance on contrast MRI^24,25^. RCC with an enhanced cystic wall may represent a transitional state between RCC and PCP. Tumors beneath the diaphragma sellae are unlikely to induce hydrocephalus^10^ even when sizable. However, if they breach the diaphragma sellae, hydrocephalus may ensue.

Regarding the immune microenvironment, this study scrutinized the expression of immune checkpoint markers, namely PD-L1 and PD-1, alongside another immunosuppressive marker, CD38 ^26,27^. Our findings revealed high PD-L1 expression on tumor cells in both PPCP and APCP, aligning with previous studies indicating elevated PD-L1 levels in tumor cells surrounding the fibrovascular core^28^. Coy et al. also proposed the potential efficacy of targeting PD-L1 and/or PD-1 as a therapeutic strategy for PCP^28^. Nevertheless, PPCP exhibiting significantly lower levels of PD-L1 expression than APCP (Fig. 4E), implying potentially varied efficacy of PD-L1 and/or PD-1 targeted therapy for PPCP compared to APCP. Conversely, CD38-positive cells were present in the stroma of both PPCP and APCP, with significantly higher levels in PPCP compared to APCP (Fig. 4F, G, H). CD38, a glycoprotein, serves as the principal catabolic enzyme for nicotinamide adenine dinucleotide (NAD+). Its association with NAD + metabolism influences various physiological processes, including infection, aging, and tumor pathogenesis. Notably, the enzymatic activity of CD38 contributes to the enhancement of tumorigenic properties within the tumor microenvironment. For instance, in hypoxic tumor microenvironments, CD38 enzymatic function fosters an immunosuppressive milieu. This information not only suggests potential differences in the focusing factors for the mechanisms of immune escape regulation in PPCP and APCP, but also highlights CD38 as a potential immunoregulatory target in PPCP.

The neutrophil marker, S100A8/A9^29^, exhibited nearly 100% expression in the non-basal layer of the tumor parenchyma in both PPCP and APCP (Fig. 5A, B). Another neutrophil marker, MPO, displayed expression in both the parenchyma and stroma of PPCP and APCP (Fig. 5C, D). S100A8/A9 expression was also observed in the stoma of both PPCP and APCP, quantified through immunohistochemistry, with higher levels observed in children compared to adults (Fig. 5F). Quantitatively, MPO expression was elevated not only in the parenchyma (Figs. 5G) but also in the stroma of PPCP compared to APCP (Fig. 5H). The S100A8/A9-positive cells identified in the stroma of PCP and the MPO-positive cells observed in both parenchyma and stroma were likely tumor-associated neutrophils. Previous studies have demonstrated that these cells played crucial roles in promoting various aspects of tumor progression, including growth, infiltration, metastasis, neovascularization, and immunosuppression^30–32^. Our findings aligned with the heightened inflammatory infiltration observed in PPCP under H&E staining, implying a more prominent involvement of tumor-associated neutrophils in PPCP. While both MPO and S100A8/A9 serve as neutrophil markers, the distinct expression patterns of these markers in PPCP suggested that the cells labeled by MPO and S100A8/A9 may belong to different subtypes of neutrophils. The up-regulation of S100A9 has been associated with the acquisition of resistance to BRAF inhibitors^33^. Greater stromal expression of S100A8/A9 in PPCP could impact the efficacy of BRAF inhibitors in treating PPCP. Our prior investigation revealed that CP with high expression of M2 macrophages, as marked by CD163, exhibited a propensity for recurrence^34^. In this study, CD163-positive cells were observed in both the parenchyma and stroma of both PPCP and APCP (Fig. 6E, F). However, APCP exhibited higher CD163 expression in the parenchyma (Fig. 6H), while CD68 expression in M1 macrophages did not differ between PPCP and APCP. Given the emerging significance of tumor-associated macrophages as a target for cancer therapy^35,36^ our future focus will be on investigating tumor-associated macrophages.

Previously, the primary treatment for PCP was surgery. In recent years, targeted therapy for the BRAF mutation has emerged with some success following its discovery^15,37–41^. As analyzed above, there is uncertainty regarding whether PPCP would derive similar benefits from targeted therapy as observed in other PCP. Given its pronounced inflammatory features, anti-inflammatory therapy may represent a viable option, either alone or in combination with targeted therapy. CP are typically managed through two surgical approaches: transsphenoidal and transcranial resection^18,42–46^. Previously, the limited understanding of PPCP has resulted in preoperative misdiagnosis and inappropriate treatment, including intraoperative management. For example, diagnosing abscesses often leads to surgical interventions involving simple incision and drainage. However, this approach can result in postoperative misdiagnosis, ultimately failing to achieve therapeutic objectives and potentially harming the patient^10^. Surgery for PCP primarily aims to minimize hypothalamic damage and prevent vision loss while preserving the patient’s quality of life after treatment^7,18,44,47^. PPCP typically resides beneath the diaphragma sellae, which acts as a natural barrier between the tumor and adjacent tissues, notably including the hypothalamus^48,49^. This positioning allows for the possibility of complete tumor resection without hypothalamic damage^10,43,49^. In our previous report, complete tumor resection was achieved in 5 PPCP patients without recurrence during a follow-up period of up to 56 months.

Due to the sporadic occurrence of PPCP cases, and despite the multi-center data collection, a prospective study design was not feasible. So, limiting the ability to perform temporal matching comparisons with APCP cases and precluding further stratified analyses.

In conclusion, our study underscored that while PPCP and APCP shared similarities in driver genes and methylation, significant distinctions existed in tumor location, histology, imaging features, proliferation, and immune microenvironment. PPCP is not merely a miniature version of APCP; therefore, it is crucial to consider the possibility of PPCP during preoperative diagnosis, especially based on distinctive imaging features. When devising therapeutic strategies, such as anti-inflammatory or potentially targeted therapies, it is imperative to account for the influence of difference in the immune microenvironment between PPCP and APCP.

## Electronic supplementary material

Below is the link to the electronic supplementary material.

Supplementary Material 1

## Data Availability

The data of this study are available from the corresponding author upon reasonable request.

## Abbreviations

CP: Craniopharyngioma
RCC: Rathke’s cleft cyst
ACP: Adamantinomatous craniopharyngioma
PCP: Papillary craniopharyngioma
APCP: Adult papillary craniopharyngioma
PPCP: Pediatric papillary craniopharyngioma
