# Supplementary material for: Comparative analysis of clinical features, methylation and immune microenvironment in pediatric and adult papillary craniopharyngiomas: results from a multicenter study
Source: Sci Rep. 2025 Jul 28;15:27422. doi: 10.1038/s41598-025-12662-8 (PMC12304257; doi:10.1038/s41598-025-12662-8)
Supplement: Supplementary file 1 — Supplementary Material 1 [file 41598_2025_12662_MOESM1_ESM.docx]

**Supplementary1. Immunohistochemistry staining procedures**

1. Baking the Slides: Put the slides into the oven, set the temperature to 72 degrees, and bake for 30 minutes.
2. Deparaffinization and Hydration: Deparaffinize the paraffin sections by immersing them into three consecutive xylene baths for 10 minutes each, followed by treatment in a graded ethanol series (absolute ethanol → 95% → 75%) for 2 minutes each. Finally, rinse thoroughly with tap water and then with distilled water.
3. **Antigen Retrieval**:Heat the EDTA (pH 9.0) working solution in a pressure cooker until boiling, then place the slides into the cooker. Once the pressure valve starts rotating, continue heating for 2.5 minutes. Allow the slides to cool to room temperature by immersing in water, and then rinse thoroughly with distilled water.
4. **Blocking Endogenous Peroxidase**:Incubate the slides in 3% hydrogen peroxide in deionized water for 15 minutes to block endogenous peroxidase activity. Wash the slides three times with PBS, 2 minutes each.
5. **Primary Antibody Incubation**:Apply the primary antibody solution to the slides and incubate overnight at 4°C. After incubation, wash the slides three times with PBS, 2 minutes each.
6. **Secondary Antibody Incubation**: Apply the secondary antibody solution to the slides and incubate at room temperature or 37°C for 20 minutes. Wash the slides three times with PBS, 2 minutes each.
7. Color Development: Develop the color using DAB solution. Prepare the DAB staining solution according to the manufacturer’s instructions, and apply it to the tissue sections. Monitor the color development under a microscope. Once the desired staining intensity is achieved, stop the reaction by rinsing with water.
8. **Counterstaining**:Rinse the slides thoroughly with tap water, then counterstain with hematoxylin for 2 minutes. Differentiate in 1% acid alcohol, and rinse with tap water to restore the blue color.
9. Dehydration and Mounting:Dehydrate the slides in a graded ethanol series (80%, 95%, absolute ethanol), 5 minutes each. Clear the slides in two changes of xylene, 2 minutes each. Finally, mount the slides with a coverslip.
